# Evaluating Phthalates and Bisphenol in Foods: Risks for Precocious Puberty and Early-Onset Obesity

**DOI:** 10.3390/nu16162732

**Published:** 2024-08-15

**Authors:** Valeria Calcaterra, Hellas Cena, Federica Loperfido, Virginia Rossi, Roberta Grazi, Antonia Quatrale, Rachele De Giuseppe, Matteo Manuelli, Gianvincenzo Zuccotti

**Affiliations:** 1 Department of Internal Medicine and Therapeutics, University of Pavia, 27100 Pavia, Italy; 2 Pediatric Department, Buzzi Children’s Hospital, 20154 Milano, Italy; virginia.rossi@unimi.it (V.R.); roberta.grazi@unimi.it (R.G.); antonia.quatrale@unimi.it (A.Q.); 3 Laboratory of Dietetics and Clinical Nutrition, Department of Public Health, Experimental and Forensic Medicine, University of Pavia, 27100 Pavia, Italy; hellas.cena@unipv.it (H.C.); federica.loperfido@unipv.it (F.L.); rachele.degiuseppe@unipv.it (R.D.G.); 4 Clinical Nutrition and Dietetics Unit, ICS Maugeri IRCCS, 27100 Pavia, Italy; m.manuelli88@gmail.com; 5 Department of Biomedical and Clinical Science, University of Milano, 20157 Milano, Italy; gianvincenzo.zuccotti@unimi.it

**Keywords:** phthalates, bisphenol, foods, precocious puberty, obesity, children, adolescents, pediatrics

## Abstract

Recent scientific results indicate that diet is the primary source of exposure to endocrine-disrupting chemicals (EDCs) due to their use in food processing, pesticides, fertilizers, and migration from packaging to food, particularly in plastic or canned foods. Although EDCs are not listed on nutrition labels, their migration from packaging to food could inadvertently lead to food contamination, affecting individuals by inhalation, ingestion, and direct contact. The aim of our narrative review is to investigate the role of phthalates and bisphenol A (BPA) in foods, assessing their risks for precocious puberty (PP) and early-onset obesity, which are two clinical entities that are often associated and that share common pathogenetic mechanisms. The diverse outcomes observed across different studies highlight the complexity of phthalates and BPA effects on the human body, both in terms of early puberty, particularly in girls, and obesity with its metabolic disruptions. Moreover, obesity, which is independently linked to early puberty, might confound the relationship between exposure to these EDCs and pubertal timing. Given the potential public health implications, it is crucial to adopt a precautionary approach, minimizing exposure to these EDCs, especially in vulnerable populations such as children.

## 1. Introduction

Endocrine-disrupting chemicals (EDCs) are compounds or mixtures of chemicals that may interact with the human endocrine system, producing site-specific effects on various organs such as the liver, pancreas, adipose tissue, and reproductive system [1,2]. These molecules may induce a span of effects beyond hormonal activity, including mitochondrial dysfunctions, oxidative alterations, epigenetic modifications, and changes in gut microbiota activities [3,4]. EDCs are widely found in personal care items, plastic containers, toys for babies, beverage cans, cosmetics, shampoo, shower gel, and several food ingredients. Importantly, since humans are often exposed to a mixture of these chemicals, analyzing health risks is even more challenging [1].

Recent research has suggested a potential connection between exposure to EDCs and the increasing prevalence of various endocrine, metabolic, and reproductive-related diseases and disorders among children [5].

Specifically, bisphenol A (BPA) (2,2-Bis (4-hydroxyphenyl) propane) [HO-C_6_H_4_-C(CH_3_)_2_-C_6_H_4_-OH] and phthalates [C_6_H_4_(CO_2_CH_2_CH(C_2_H_5_)(CH_2_)_3_CH_3_)_2_] are particularly concerning as a growing number of studies have shown their potential role in promoting obesity and other metabolic disorders, threatening the function of the reproductive system [6,7,8].

Humans are exposed to EDCs throughout their entire lifespan: from the gestational period and early life through to adulthood. Several studies have documented that vulnerable populations, such as pregnant women, infants, and children, are notably sensitive to these compounds. Indeed, in their cross-sectional research, Zhou and colleagues reported a positive association between phthalate exposure and negative cardiometabolic health outcomes in a cohort of 115 pregnant women, particularly concerning inflammation and the lipid biogenesis profile [9]. Moreover, Ouidir and colleagues demonstrated that exposure to mono-n-butyl phthalate (MnBP) at 12 months of life is linked to a higher body mass index (BMI), weight, and head circumference in infants at 36 months of age. This suggests that exposure to phthalates in early life may be associated with increased weight and BMI in early childhood, leading to a higher risk of obesity and other correlated disorders later in life [10].

The National Health and Nutrition Examination Survey (NHANES) survey, which considers the period between 2003 and 2014, registered a suggestive positive association between the urinary levels of phthalates metabolites and metabolic syndromes in a cohort of children and adolescents [11]. Higher urinary levels of MnBP were associated with higher odds of dyslipidemia in males. Additionally, several studies have indicated that phthalates can both disrupt puberty during adolescence and influence metabolism through mechanisms involving estrogenic activity and the activation of peroxisome proliferator-activated receptors (PPARs), which play roles in adipocyte differentiation and insulin sensitivity [12].

BPA exposure is associated with obesity in both animal and human studies. It disrupts metabolic function, leading to conditions such as dyslipidemia, elevated leptin levels, hyperglycemia, and insulin resistance [13]. Elevated urinary BPA levels have been observed in children and adolescents with obesity, and high BPA levels in young girls are associated with a higher risk of obesity [14]. Considering the significant role of obesity in triggering puberty, it is reasonable to speculate that BPA could indirectly impact puberty by disrupting metabolism and endocrine balance [15].

Recent scientific results indicate that diet is the primary source of exposure to EDCs due to their use in food processing, pesticides, fertilizers, and migration from packaging to food, particularly in plastic or canned foods [16]. Although EDCs are not listed on nutrition labels, their migration from packaging to food could inadvertently lead to food contamination, affecting individuals by inhalation, ingestion, and direct contact [17].

The aim of our narrative review is to investigate the role of phthalates and BPA in foods, assessing their risks for precocious puberty (PP) and the early onset of obesity, which are are two clinical entities that are often associated and that share common pathogenetic mechanisms. The role of EDCs in the close correlation between PP and obesity cannot be excluded. An in-depth examination of the topic can be useful in promoting preventive strategies and ensuring healthier developmental outcomes.

## 2. Methods

We conducted a narrative review [18,19] to discuss the role of diet in exposure to phthalates and bisphenol and to investigate how their presence in foods may be a risk factor for the development of PP and early-onset obesity. We undertook an extensive literature search on PubMed, focusing on English-language articles published in the last 25 years, up to June 2024. The types of publications examined included original research articles, systematic reviews, meta-analyses, and longitudinal studies. Case reports, letters, and editorials were excluded. Research was found using the following keywords (alone and/or combined): nutrition, diet, phthalates, bisphenol, precocious puberty; early onset of obesity; pediatric population; childhood; and children.

Initially, we evaluated a larger number of articles, refining our selection by screening abstracts and then performing detailed full-text evaluations of relevant studies. Each article was rigorously reviewed to enable a critical analysis. Additionally, the reference lists of all articles were checked to identify relevant studies. The draft manuscript was reviewed by all co-authors and received final approval from everyone.

## 3. Results

### 3.1. Selection Process

**Figure 1:**
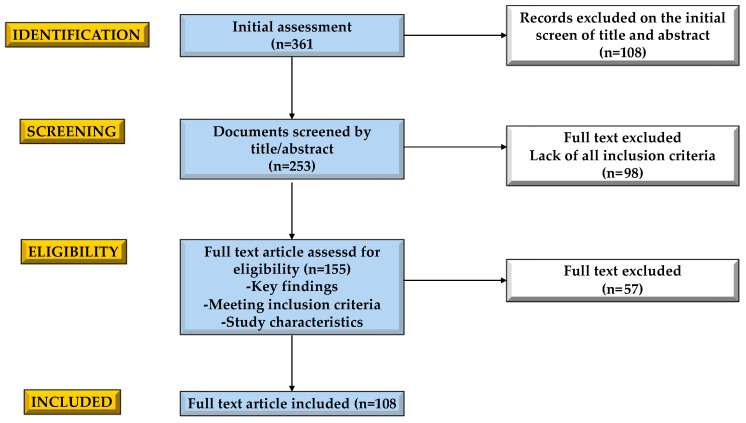
Flowchart of criteria for study selection.

The manuscript selection process is outlined in Figure 1.

### 3.2. Define Phthalates and BPA Including Their Chemical Properties and Sources

#### 3.2.1. Phthalates

The global demand for plasticizers is increasing. In 2017, an estimated 3.07 million tons of di(2-ethylhexyl) phthalate (DEHP) were consumed. While certain phthalates are restricted in Europe, regulations outside the European Union are less severe, allowing these compounds to be included in various products sold even on the European market, including food industry products from non-EU countries [20,21]. Industries have begun using phthalate substitutes like DINCH, a non-phthalate plasticizer that has replaced DEHP in different items. Initial studies suggest a negative association between urinary DINCH levels in children and their BMI z-scores, body fat percentage, and waist circumference z-scores [22]. However, further research is needed to better understand the health effects of these substances. 

Phthalates, including DEHP, dibutyl phthalate (DBP), diethyl phthalate (DEP), diisononyl phthalate (DiNP), and diisodecyl phthalate (DiDP), are chemical compounds commonly used as plasticizers in the plastic industry, particularly to create polyvinyl chloride (PVC) [23,24]. They are currently included in many products such as adhesives, air fresheners, detergents, food packaging, and clothing. For instance, while the phthalate levels in fruits and vegetables are much lower than those in processed foods, some studies suggest that in the case of labeled fruit, phthalates held in the adhesive can migrate to the entire pulp of the fruit, particularly if the label is directly attached to the fruit [25].

The metabolism of phthalates involves two stages: initial hydrolysis after cellular absorption followed by conjugation to convert them into hydrophilic glucuronide conjugates [26]. To date, secondary metabolites exhibit health effects as well [27]. Studies on animal models have shown that MEHP, a secondary metabolite of DEHP, impacts reproductive functions. The lipophilic nature of these compounds suggests that diet is one of the main routes of human exposure [28]. Since diet is the primary exposure route, Table 1 lists commonly consumed foods and packaging containing phthalates.

**Table 1:** List of common foods containing phthalates.

Reference	Matrix	Phthalate	Average Concentration
da Costa JM, et al., 2023 [29]	Baby food	DMP	ND–0.2 (ND) (μg/kg)
		DEP	ND–1.6 (0.1) (μg/kg)
		DiBP	0.1–16.0 (2.7) (μg/kg)
		DnBP	0.1–32.0 (1.3) (μg/kg)
		BBP	ND–16.0 (2.1) (μg/kg)
		DEHP	ND–67.0 (22.0) (μg/kg)
		DCHP	ND–1.8 (ND) (μg/kg)
		DnOP	ND–3.0 (0.2) (μg/kg)
	Cereals, mixed	DiBP	2.41 (μg/kg)
		BBzP	<8.73 (μg/kg)
		DEHP	41.4 (μg/kg)
	Popcorn, microwave	DiBP	39.8 (μg/kg)
		DBP	208 (μg/kg)
		BBzP	34.7 (μg/kg)
		DEHP	284 (μg/kg)
	Chocolate bars	DiBP	<15.2 (μg/kg)
		DBP	<14.0 (μg/kg)
		BBzP	5.04 (μg/kg)
		DEHP	135 (μg/kg)
Wu CG, et al., 2014 [30]	Cola	DMP	105 (µg L^−1^)
		DEHP	1123 (µg L^−1^)
Wu CG, et al., 2014 [30]	Fruit juices	DEHP	22–126 (µg L^−1^)
Nanni N et al., 2011 [31]	Extra virgin olive	DEHP	1134 (µg L^−1^)
		DINP	1722 (µg L^−1^)
		DBP	90 (µg L^−1^)
Fierens T et al., 2013 [32]	Milk, milked by hand	DEHP	<60 (µg L^−1^)
		DIBP	29 (µg L^−1^)
		DBP	<15 (µg L^−1^)
		BBP	<10 (µg L^−1^)
Fierens T et al., 2013 [32]	Milk, milked by machine	DEHP	123.5(µg L^−1^)
		DIBP	15.1 (µg L^−1^)
		DBP	ND
		BBP	14.3 (µg L^−1^)
Hou H et al., 2021 [25]	Apple pulp with label	Long chains PAEs	0.08 (mg/kg)
		Short chains PAEs	0.16 (mg/kg)
	Avocado with label	Short chains PAEs	0.83 (mg/kg)
		Long chains PAEs	1.04 (mg/kg)

ND = not detectable.

#### 3.2.2. Biphenol A

The global market for BPA in 2022 was estimated to be around 5600 tons. Projections for 2032 suggest a 3.51% increase, reaching 8000 tons with Europe and North America being the highest BPA consumers [33].

Following ingestion, BPA is generally absorbed through the digestive tract and partially digested by the intestinal microbiota, which may both directly and indirectly interact with EDCs molecules which can disrupt gut microbiota homeostasis, threatening intestinal barrier permeability and leading to dysbiosis [34]. Subsequently, EDCs are metabolized in the liver mainly by glucuronidation and excreted by the kidneys. BPA-glucuronide is the secondary metabolite of BPA and has a higher bioactivity than the original molecule as well as phthalate secondary metabolites [35].

Recent studies have shown that while BPA is mainly detected in urine samples, it has also been found in the placenta and breast milk, leading to increased exposure to BPA during early life, both directly to the newborn and indirectly through maternal exposure [4].

To date, sixteen structural analogs of BPA have been identified in substitution with BPA itself, particularly in the manufacturing of baby items [36]. However, the primary concern is the limited availability of data about their effects on human health. Recent studies have pointed out that these alternative compounds may not be as harmless as originally believed. For example, bisphenol S (BPS) and bisphenol AF (BPAF) have displayed similar or even more significant side effects compared to BPA [37]. Specifically, BPS, which is used as a substitute for BPA in the production of epoxy resins, thermal paper, and baby bottles, has been found to cause adverse metabolic effects [38].

As for phthalates, food processing and diet are the primary routes for bisphenol release due to the contaminated environment and the migration from packaging to food or beverages. Heat and pH can accelerate this process, allowing BPA to move into canned and processed foods, as well as high-fat foods. Table 2 shows BPA concentrations in food products.

Given their lipophilic characteristics and use in food containers, these molecules accumulate in the adipose tissue or fat. Consequently, a diet rich in fresh foods and with a lower number of processed foods such as the Mediterranean Diet will limit exposure to these molecules. However, despite the European Food Safety Authority (EFSA) having established a lower acceptable daily intake of BPA at 0.2 ng/kg (around 20,000 times lower than before), ongoing human exposure raises questions over cumulative health hazards and co-exposures to combinations of phthalates and bisphenols [39].

### 3.3. The Role of Diet in Exposure to Phthalates and Bisphenol

Animal-derived foods, including beef, pig, and chicken, are noteworthy sources of high-molecular-weight phthalates (HMWPs) and BPA due to their processing and packing life cycle processes [40]. HMWPs, which are barely lipophilic, can accumulate in fat-rich ingredients. International food monitoring research exhibits high levels of HMWPs, particularly DEHP and BPA, in meat and meat products [41], suggesting animal products as potential sources of DBP, DEP, and di-iso-butyl phthalate (DiBp). EDCs have been also detected in aquatic environments all over the world because of increasing pollution, affecting mainly aquatic species such as mussels and fish [42]. Notably, an excessive intake of canned or ready-to-eat fish, which is frequently wrapped in plastic, poses an important health risk, especially in vulnerable populations, such as pregnant women, children, and adolescents. In a recent study conducted in a cohort of 585 Spanish adolescents, the authors demonstrated that canned tuna was a major source of BPA in both females and males’ diets [41]. In contrast, as globally recommended by existing guidelines, minimally processed food such as fruits and vegetables, are the best choices to mitigate exposure to these molecules as well as correlated health risks [40].

For instance, recent investigations demonstrate detectable levels of EDCs also in ready-to-eat and frozen veggies, highlighting that the best solution is to use fresh foods that do not require long industrial manufacturing processes [43]. Nowadays, access to packaged and processed foods has increased, overlapping with the rise in childhood obesity rates. Due to the nutrition transition and globalization, even low-income countries and households have gained easier access to less expensive and lower-quality foods, showing an increase in overweight and obesity rates starting from early life [44,45]. Unhealthy lifestyles and dietary behaviors established during early life persist into adulthood and can lead to an increased risk of developing obesity as well as cardiometabolic and endocrinological comorbidities later in life [46]. The excessive consumption of dense energy drinks, ultra-processed snacks, and fast foods, contributes to health risks as well as to BPA and phthalates exposure [47].

According to Buckley and colleagues’ results, consuming more than 10% of energy from ultra-processed foods is associated with an 8.0% (95% CI: 5.6%, 10.3%) rise in urinary levels of phthalates metabolites such as mono-3-carboxypropyl phthalate (MCPP), mono- (carboxyisononyl) phthalate (MCNP), and mono-(carboxyisoctyl) phthalate (MCOP), moreso in adolescents than in the adults population. In particular, sandwiches, hamburgers, French fries and other packaged chips, and ice cream, were also tied to higher urinary levels of EDCs [48]. These results were confirmed by other research in which participants who reported a higher number of meals at restaurants or fast-food places, and frozen pizza in the 30 days before the dietitian interview, registered higher urinary levels of BPA [49]. Furthermore, in a pilot study conducted in northern Italy, the association between the consumption of sauces and dressings stored in plastic containers and phthalate urinary levels has been found (*p* = 0.037) [50].

On the other hand, consuming fresh foods was associated with lower levels of the same metabolites and BPA and F [48]. Furthermore, adolescents with higher exposure to BPA exhibited an increased risk of cardiometabolic biomarkers. Those with high BPA urinary levels registered also higher BMI Z-scores (0.68 kg/m² in high exposure, 0.39 kg/m² and 0.52 kg/m² in medium and low exposure, *p* = 0.008), a larger waist circumference (cm) (76.2 vs. 73.7 and 74.9, *p* = 0.026), and greater body fat levels (16.3 kg vs. 13.8 kg and 14.6 kg; *p* = 0.002). Also, insulin levels (µg/mL) were increased in adolescents highly exposed to BPA (14.1 vs. 12.7 and 13.1; *p* = 0.039), as well as triglyceride blood levels (mg/dL) (72.7 vs. 66.1 and 66.5; *p* = 0.030). Moreover, cardiometabolic risk was significantly correlated with higher BPA exposure after adjustments (OR: 2.55; 95% CI: 1.41, 4.63) [51]. Although fresh and minimally processed foods are associated with lower urinary levels of EDCs in the general population, the literature has been inconsistent with analyses related to the levels of endocrine molecules and dietary patterns as a whole [52]. For example, using the Healthy Eating Index (HEI), van Woerden and colleagues reported that high scores on the HEI and Mediterranean and vegetarian dietary patterns were associated with lower odds of a high BPA concentration (OR: 0.59, and 0.60, respectively) [49]. However, other studies are further needed to better understand how diet quality may mediate EDC exposure.

### 3.4. The Influence of Nutrition on the Risk Factor for Precocious Puberty and Its Relation to Early-Onset of Obesity

Puberty marks a milestone in human development [53,54]. It is characterized by a series of biological processes that lead to full reproductive capacity [54,55]. Its timing occurs depending on the interaction between hormones, central neurotransmitters, and environmental factors, which collectively activate the hypothalamic–pituitary–gonadal (HPG) axis [53,54,56].

A diagnosis of PP is established when secondary sexual characteristics manifest before the age of eight in females and before the age of nine in males [54,57,58]. In females, these secondary sexual characteristics include the development of breasts, the growth of pubic and axillary hair, and pelvic enlargement. In males, they include testicular enlargement, penile enlargement, and the development of axillary hair, seminal vesicles, and the prostate gland [54,57,58]. Based on its pathogenesis, PP is categorized into central (gonadotropin-releasing hormone-dependent) and peripheral (gonadotropin-releasing hormone-independent) [54,57]. The current literature indicates an increasing prevalence of this condition [59], which is associated with various health implications [60]. Specifically, it leads to the early fusion of the bony epiphyses, resulting in short adult stature; it also increases the risk of arterial hypertension, diabetes, obesity, and infertility in adulthood, as well as psychological issues [53]. Predisposing factors have been identified in previous studies [54,60].

Dong et al. [61] conducted a study to explore the related factors of PP in children. They highlighted several statistically significant risk factors for children’s PP, including female gender, bone age greater than 10 years, lack of daily physical activity, mother’s menarche time earlier than 12 years old, living in a chemical industry area, frequent consumption of nutritional supplements and a high-protein diet, scarce sleep time relative to one’s age, and an increase in specific hormones, i.e., E2, LH, and leptin.

The increased incidence of PP appears to be related to improved living standards and the influence of environmental factors. It is important to highlight what these factors are to reverse this trend [54]. In terms of nutritional risk factors, artificial feeding after birth, high sweet/snack consumption, and fried food diets seem to be correlated [54,62,63].

Numerous studies involving various populations have reported that a high intake of vegetables, fruit, vegetable proteins, and lean meat acts as a protective factor against PP [64,65]. On the other hand, the impact of milk consumption remains controversial; some studies indicate a negative correlation between milk intake and the age of menarche [66], while others find no correlation [67].

As stated before, Gu et al. [68] pointed out that, after adjustment for age and BMI scores, a diet high in red meat, fruit, and eggs is significantly positively associated with PP.

In the last few decades, the relationship between obesity and CPP has been highlighted, as several studies have shown that especially girls who experience early puberty onset are more likely to accumulate excessive body weight.

Research has aimed to understand how obesity is connected to early puberty [69]. The most supported theories focus on the hormonal role of fat cells and adipose tissue, especially insulin resistance, the secretion of adipokines, and the activity of aromatase in the body. As in individuals with obesity, the levels of adiponectin, insulin, ghrelin, and leptin are disrupted, and the same molecules affect the regulation of the hypothalamic–pituitary–gonadal axis (HPG), impacting the timing of puberty [70,71]. Since eating habits have an impact on preventing childhood obesity, researchers have also investigated adherence to dietary patterns during puberty. An observational study in the US showed that girls who followed the Mediterranean Diet tended to experience puberty later than those who did not [72]. For example, results from Moslehi’s research have shown that replacing 10 g of animal protein with veggie protein was associated with a lower risk of PP [16% (95%CI: 5–25%; *p* = 0.006)] [73]. A similar delay has been seen in girls who ate fruits and vegetables rich in antioxidants and flavonoids. Moreover, in Chinese females, a diet rich in vegetables and vegetable proteins were significantly negatively associated with early puberty (OR = 0.78, 95% CI: 0.63–0.97) [68]. In contrast, excessive consumption of animal proteins was linked with PP (OR = 1.36, 95% CI: 1.09–1.69), after adjusting for age and body mass index scores [74].

Potential pathogenetic mechanisms linking the consumption of these foods to the development of PP involve hormonal patterns, including IGF-1 and adipokines, as well as micronutrients such as iron and zinc [41,75]. These micronutrients, which are present in red meat and essential for supporting pregnancy and fetal health, may be associated with the onset of puberty [76].

Moreover, research has identified several pollutants that influence pubertal growth and, among these, phthalates and bisphenols, both categorized as EDCs, are particularly significant. As detailed in the next sections, phthalates may influence pubertal development and adiposity through estrogenic activity and the activation of peroxisome proliferator-activated receptors (PPARs) [76].

**Figure 2:**
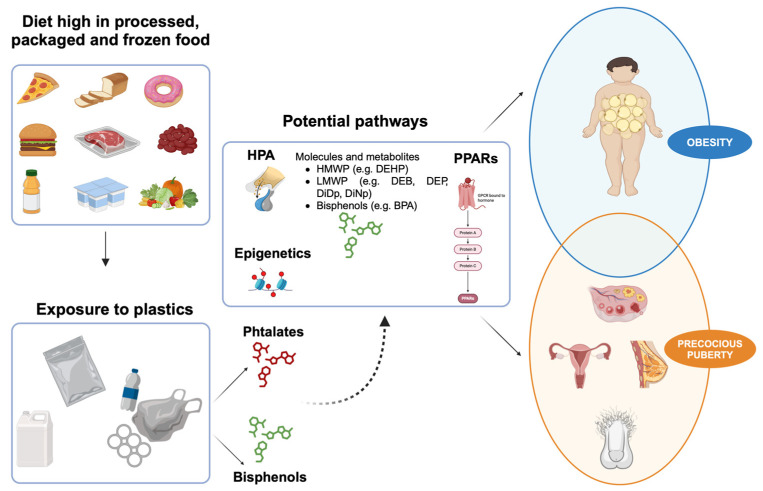
The interaction between diet, phthalates, and bisphenols with precocious puberty and early onset of obesity. Created by Biorender.com®. DEHP = Di(2-ethylhexyl) phthalate; DEP = diethyl phthalate, DiDP = diisodecyl phthalate; DiNP = diisononyl phthalate; HHWM = high-molecular-weight phthalates; HPA = hypothalamic-pituitary-adrenal; LHWM = low-molecular-weight phthalates; PPARSs = Peroxisome proliferator-activated receptors.

Figure 2 shows the interaction between diet, phthalates, and bisphenols with precocious puberty and early onset of obesity.

### 3.5. Impact of Bisphenol and Phthalates in Foods

Despite the numerous mechanisms through which BPA affects the human body still not being clear, its estrogen-mimicking properties have been extensively studied. As a synthetic estrogen, BPA competes with endogenous estradiol for binding to estrogen receptors [77,78,79,80]. Its ability to act as an estrogen disruptor by interfering with nuclear estrogen receptors may explain its dual role in triggering early puberty and compromising fertility. This dual functionality, acting as either an agonist or antagonist through traditional or alternative pathways, contributes to its impact [77]. Additionally, BPA disrupts steroid hormone activity by antagonizing the effects of androgens and thyroid hormones [77,81,82]. Numerous studies suggest that BPA may play a significant role in the development of various genital and reproductive disorders. These disorders include alterations in the male and female reproductive tracts, infertility in both sexes, the early onset of puberty, cancers, polycystic ovary syndrome, and irregular menstrual cycles [77,83,84,85,86,87].

Therefore, BPA exposure can lead to PP due to its estrogen-mimicking properties, which stimulate the GnRH pulse generator through a positive feedback loop, resulting in elevated levels of LH and FSH secretion, despite BPA’s relatively weak estrogenic activity. Animal research has shown that BPA affects the hypothalamic–pituitary–gonadal axis by influencing the sexual differentiation of the brain, increasing GnRH release, and disrupting the LH surge triggered by estradiol [77,85].

Weight status is also related to pubertal age, as environmental pollutants may increase the risk of being overweight. The decline in the age of puberty could be partly explained by excess weight resulting from exposure to certain environmental pollutants [1,2]. Exposure to EDCs may adversely affect adipose tissue function and metabolism, potentially resulting in childhood high weight and obesity [3]. The mechanisms through which phthalates exert their effects are multifaceted and may encompass estrogenic activity and the activation of the nuclear transcription factor peroxisome proliferator-activated receptor-γ (PPAR-γ), which play roles in adipocyte differentiation and insulin sensitivity. The effects of phthalates on adipose tissue are influenced by factors such as biological species, cell type, and dosage, which can activate different PPAR isoforms.

**Table 2:** Bisphenol A concentrations in food products.

Reference	Matrix	Bisphenol	Average Concentration
Lee J. et al., 2019 [88]	Baby food for 15-month-old children	BPA	5.09 (ng/g)
Russo G. et al., 2019 [75]	Non-canned fruits, dried fruits, nuts, and seeds	BPA	Min–Max (μg kg^−1^) 0.105–2.130
Russo G. et al., 2019 [75]	Canned tuna	BPA + BPs	Min–Max (μg kg^−1^) 6.3–187.0
Schiano ME. et al., 2023 [76]	Canned legumes	BPA	1.51–21.22 (ng/mL)
Schiano ME. et al., 2023 [76]	Sliced bread (plastic packaging)	BPA	ng/g (SD) 1.20 (0.3)
Schiano ME. et al., 2023 [76]	Salted snacks (plastic packaging)	BPA	ng/g (SD) 25.45 (23.54)
Robles-Aguilera V. et al., 2021 [89]	Ham (plastic packaging)	BPA	ng/g (SD) 6.6 (3.4)
Schiano ME. et al., 2023 [76]	Cake (not packaged)	BPs	ng/g (SD) 1.7 (0.7)
Robles-Aguilera V. et al., 2021 [89]	Rice (plastic packaging)	BPs	ng/g (SD) 3.3 (1.4)

SD = standard deviation; BP = bisphenol.

### 3.6. Studies Exploring the Impact of Phthalates and BPA in Precocious Puberty and Early-Onset Obesity

#### 3.6.1. BPA and Precocious Puberty

Epidemiological studies have linked BPA exposure to early puberty, such as premature pubarche and menarche in girls of normal weight [90,91], premature thelarche [92,93], and a higher incidence of idiopathic central precocious puberty (ICPP) in females [63].

Wolff et al. conducted a notable study on the relationship between environmental–dietary phenols, including BPA, and pubertal development in girls. Their initial research in 2008 involved 192 multiethnic 9-year-old girls in New York City, where urinary BPA levels were measured alongside other contaminants such as phytoestrogens (as genistein C_15_H_10_O_5_ and daidzein C_15_H_10_O_4_), dichlorodiphenyldichloroethylene (C_14_H_8_Cl_4_), and byphenil (C_12_H_10_). Even after adjusting for BMI scores, no discernible correlation was found between urinary BPA levels and the progression of puberty [77,94].

In 2010, Wolff and colleagues broadened their research to encompass 1151 American girls aged 6 to 8 years from the BCERC study, monitoring their development through the puberty stages. They examined the impact of various environmental chemicals, including BPA, on pubertal timing over several years [77,95]. A year later, they observed a decrease in the percentage of girls showing breast development (30%) or pubic hair growth (22%), which may be attributed to the younger age group studied. Urine samples collected at both enrollment and one year later were analyzed, but similar to their previous findings, there was no evidence of a connection between BPA levels detected in urine and the observed stage of puberty [95].

A subsequent study in 2015 by the same group involved 1239 girls from the BCERC cohort, examining the age of first puberty onset and its correlation with urinary phenol levels, including BPA. Despite observing pubertal onset in a majority of the girls, no statistically significant link was found between BPA levels and early puberty [96].

In 2012 Buttke, Sircar, and Martin investigated 461 American girls aged 12–16 years from the NHANES study (2003–2008), finding no significant link between urinary BPA levels and age at menarche, despite considering BMI scores and ethnicity as potential modifiers [77,97].

Studies by Yum et al., Lee et al., and Han et al. among Korean populations also did not support a significant link between BPA levels and precocious puberty [77,98,99,100]. Similarly, Buluş et al. [101] found no significant differences in urinary BPA levels among Turkish girls with idiopathic central or peripheral precocious puberty compared to controls, suggesting other environmental factors may influence early puberty [77,101].

Similarly, Frederiksen et al. conducted a European study involving 129 Danish children and adolescents aged 6 to 21 which also found no correlation between urinary BPA and pubertal status [24,77,102].

However, in a subsequent longitudinal study that included both male and female subjects, the appearance of pubic hair was not altered in most exposed girls, whereas earlier pubarche was demonstrated in most exposed boys, who also exhibited higher testosterone levels and lower adrenal hormones [103]. In a small multicenter cross-sectional study, Lomenick et al. [104] concluded that exposure to phthalates does not correlate with earlier puberty in female children, contrasting with the study by Zhang et al. [12].

Jung et al. [105] did not find significant differences in urinary BPA levels between girls with central precocious puberty (CPP) and pubertal controls. Interestingly, they noted that the pre-pubertal group had higher BPA levels than the CPP group, hypothesizing that lower urinary BPA levels in girls with CPP might be due to increased excretion of BPA during puberty rather than reduced exposure. This finding contrasts with previous studies, suggesting that urinary BPA concentrations are influenced by both exposure levels and excretion rates.

In 2020, Bigambo et al., through a meta-analysis of nine studies involving 4737 girls, identified a significant link between exposure to 2,5-dichlorophenol and earlier puberty, with an effect size of 1.13. However, they found no significant association between early puberty and exposure to other phenolic chemicals like bisphenol A, triclosan, and benzophenone 3 [106].

Conversely, McGuinn et al. analyzed data from the NHANES study (2003–2010), focusing on 987 girls aged 12–19 years. They observed delayed menarche in girls with moderate urinary BPA levels compared to those with the lowest concentrations, although after adjusting for confounders, this association did not reach statistical significance [77,107]. Despite aligning with findings from other research, one consistent outcome was the discovery of elevated urinary BPA levels among overweight girls, correlating with an increased likelihood of experiencing early menarche [77,108].

Durmaz et al. observed significantly higher urinary BPA levels in Turkish girls with idiopathic central precocious puberty compared to controls but found no correlation with serum hormone levels (serum LH, FSH, and estradiol) [77,109]. Supornsilchai et al. found higher urinary BPA levels in Thai girls with precocious puberty, particularly among obese individuals, although BPA levels were not associated with hormone levels. They also noted that obese or overweight girls showing signs of pubertal development exhibited higher urinary BPA levels compared to both normal-weight girls and overweight or obese girls without signs of pubertal development [77,110].

In 2018, Chen et al. conducted a study in Shanghai, China, involving 136 girls aged 6 to 9 years diagnosed with idiopathic central PP, along with 136 age- and BMI-matched controls. They found that girls with the highest urinary BPA concentrations were significantly more likely (9.08 times) to have ICPP compared to those with the lowest concentrations. The study also observed a modest negative correlation between urinary BPA levels and peak FSH levels in the ICPP group, suggesting that BPA exposure may contribute to ICPP by affecting FSH levels [63].

Three years later, the same group conducted a case-control study with 121 girls aged 2–10 years diagnosed with precocious puberty. In a multivariate logistic regression analysis, bisphenol S tetrabromobisphenol A and bisphenol-FL (substitutes for BPA), measured in urine specimens, were significantly associated with a higher risk of precocious puberty. This suggests that substitutes for BPA may also be linked to an increased risk of precocious puberty in girls [90].

In another study, Lee et al. (2021) investigated peripheral precocious puberty in Korean girls and noted slightly higher urinary BPA levels, although these were not statistically significant, along with elevated levels of various sex steroids [77,111].

In 2024, Huynh et al. investigated the link between BPA and precocious puberty in children in Ho Chi Minh City and found higher urinary BPA levels in children with precocious puberty compared to controls, suggesting a potential association between these chemicals and early puberty onset [112].

While numerous studies have been conducted on females, research on male samples remains limited.

Ferguson et al. examined prenatal and postnatal exposure to BPA and phthalates in 113 boys aged 8 to 14 years, respectively, through analysis of maternal urine samples collected during the third trimester and urine samples from offspring born to those mothers. They found no significant correlation between prenatal BPA exposure and sex hormone levels in boys. However, BPA exposure during childhood was associated with higher Sex Hormone-Binding Globulin levels and lower total and free testosterone levels. Prenatal BPA exposure was linked to an earlier onset of adrenarche, potentially due to its anti-androgenic effects [77,113].

Wang et al. examined 671 Chinese boys aged 9 to 18 years to investigate the relationship between urinary BPA levels and pubertal development. They observed that moderate BPA exposure was associated with an earlier onset of pubic hair development, while higher BPA levels correlated with delayed final stages of genital development. The study suggested that BPA exposure might accelerate pubarche and adrenarche but hinder genital maturation in boys [77,114]. Notably, BMI did not seem to influence the association between BPA exposure and pubertal development in boys, which contrasts with findings in girls [77,115,116].

Overall, while some studies suggest a potential link between BPA exposure and early puberty, others do not find significant associations. This inconsistency in findings can be attributed to various factors, including differences in study design, population characteristics, exposure assessment methods, and confounding variables.

In Table 3, the studies on the impact of phthalates and BPA on PP and early onset of obesity are listed.

#### 3.6.2. Phthalates and Early Obesity

The national study Puberty Timing and Health Effects in Chinese Children (PTHEC) established an association between exposure to environmental phthalate esters (PAEs) and metabolic alterations. Mass spectrometry analysis, used to measure various PAE monoesters (MMP, MEP, MBP, MEHP, MEOHP, and MEHHP) in urine samples, revealed a metabolomic profile characterized by elevated levels of markers associated with altered arginine and proline metabolism and fatty acid re-esterification [117]. These results suggest a contributory role of EAPs in the development of high weight and obesity among school-age children. Notably, MEHP was specifically linked to visceral obesity, as indicated by waist circumference and waist-to-height ratio measurements, even in healthy individuals with normal weight [117].

Stahlhut et al. [118] demonstrated that waist circumference (WC) was associated with elevated levels of four phthalates (MBzP, MEHHP, MEOHP, and MEP). Hatch et al. [119] highlighted that BMI scores and WC showed a significant positive correlation only with MEP in adolescent females, while it was insignificant in adult women.

Significant associations with obesity, elevated BMI, and waist circumference were also documented in children according to studies by Golestanzadeh et al. [120] and Zarean et al. [121]. Zarean et al. specifically noted significant associations between weight and BMI scores, although these associations showed inconsistent trends between girls and boys [121,122].

Several systematic reviews have assessed the correlation between phthalate exposure and obesity. Goodman et al. conducted the first systematic analysis of the association between phthalates and obesity, finding little agreement both between studies and within studies regarding any phthalate metabolite and any indicator of high weight or obesity [1]. A recent systematic review reported a positive association between phthalate exposure and obesity in children during early development (≥2 years) and adults [123,124].

Buser MC [125] and her team analyzed data from the NHANES spanning from 2007 to 2010. Their findings revealed that LMWP metabolites (MnBP, MEP, and MiBP) were linked to obesity among male children and adolescents, while HMWP metabolites were associated with increased obesity risk in adults. Separately, Dong et al., 2022 [126], conducted a nested case-control study in China, demonstrating that childhood exposure to PAEs significantly elevated the risk of high weight and obesity, showing a dose–response relationship, particularly notable in girls [127]. The study by Wang et al. [114] demonstrated that urinary concentrations of MEP and MiBP were positively associated with the risk of childhood abdominal obesity among Chinese students aged 7 to 10 years. These findings suggest that widespread exposure to PAEs may be a major contributor to the high prevalence of abdominal obesity in Chinese students. Additionally, they found that the concentration of several phthalate metabolites in students with abdominal obesity was significantly higher than in students without abdominal obesity [128]. A meta-analysis of DEHP exposure and obesity-related outcomes in rodents by Wassenaar et al. [129] found a nonsignificant association between early-life exposure to DEHP and body weight. We believe that there are three main reasons for these conflicting findings: phthalates may not induce obesity as hypothesized, the studies had methodological limitations, or there were confounding factors that influenced the results. Finally, a 2023 study by Li et al. [127] highlighted that diet and physical activity, rather than phthalate metabolites, were associated with childhood obesity.

In Table 3, the studies on the impact of phthalates and early-onse obesity are listed.

**Table 3:** Studies exploring the impact of phthalates and bisphenol A (BPA) in precocious puberty and early-onset obesity.

BPA and Precocious Puberty				
Authors	Sample	Age	Type of Study	Main Results
Wolff et al., 2008 [94]	192 multiethnic girls	9 years old	Prospective cross-sectional study	No correlation between urinary BPA levels and puberty progression
Lee et al., 2009 [99]	30 patients (29 girls and 1 boy) with idiopathic CPP + 30 healthy controls	8.6 ± 0.9 vs. 7.8 ± 1.1 years old	Case-control study	Slightly higher urinary BPA levels in girls with peripheral precocious puberty
Wolff et al., 2010 [95]	1151 American girls	6–8 years old	Longitudinal study	No correlation between urinary BPA levels and puberty progression
Buttke et al., 2012 [97]	461 American girls	12–16 years old	Cross-sectional study	No significant link between urinary BPA levels and age at menarche
Frederiksen et al., 2013 [102]	129 Danish children and adolescents	6–21 years old	Cross-sectional study	No correlation between urinary BPA levels and puberty progression
Zhang et al., 2015 [12]	430	6–14 years	Cross-sectional study	Subtle effects of phthalate metabolites associated with pubertal onset and progression. MnBP exposure may be associated with delayed pubic hair development in boys, while MnBP, MMP, MEP, and MEHP exposure may be associated with breast development onset, and MEHP metabolites may be associated with a speedup in breast development and an earlier menarche onset in girls
Mouritsen et al., 2013 [103]	168	5–10 years	Longitudinal study	High exposure to DBP was associated with earlier age at pubarche in boys. In girls, no associations between phthalate exposure and age at pubertal milestones were observed
Lomenick et al., 2010 [104]	56	7 years	Cross-sectional study	Phthalates may be associated with certain other toxicities in humans; our study suggests that their exposure is not associated with precocious puberty in female children
Yum et al., 2013 [98]	150 Korean girls with ICPP + 90 healthy controls	6–12 years old	Case-control study	No significant association between BPA levels and precocious puberty
Lee et al., 2014 [111]	42 Korean girls with ICPP + 40 with IPPP + 32 healthy controls	8.7 ± 1.0 vs. 8.4 ± 0.7 vs. 8.5 ± 0.9	Case-control study	Non-statistically significant slightly higher BPA levels in ICPP and IPPP than controls
Ferguson et al., 2014 [113]	250 boys	8.10–14.4 years old	Prospective cohort	No association between prenatal BPA exposure and sex hormone levels in boys
Wolff et al., 2015 [96]	1239 Black or Hispanic girls	6–8 years old	Prospective longitudinal cohort study	No statistical link between urinary BPA levels and puberty progression
McGuinn et al., 2015 [107]	987 American girls	12–19 years old	Cross-sectional study	No significant association was found between urinary BPA levels and earlier menarche. Delayed menarche was found in girls with moderate urinary BPA levels
Supornsilchai et al., 2016 [110]	29 Thai girls with ICPP + 12 with early puberty + 47 healthy girls	7.44 ± 1.03 years old	Case-control cross-sectional study	Higher urinary BPA levels in obese girls with precocious puberty
Buluş et al., 2016 [101]	42 Turkish girl with ICPP + 42 with IPPP + 50 healthy controls	7.4 ± 0.6 years old	Case-control study	No significant differences in urinary BPA levels in girls with ICPP-IPPP
Wang et al., 2017 [114]	671 Chinese boys	9–18 years old	Cross-sectional study	Association between peripubertal BPA exposure and earlier pubertal onset, but delayed pubertal progression in boys
Durmaz et al., 2018 [93]	28 Turkish girls with ICPP + 25 healthy girls	4–8 years old	Case-control cross-sectional study	Higher urinary BPA levels in girls with CPP, no correlation with hormone levels
Chen et al., 2018 [63]	285 Chinese girls with ICPP and 136 healthy controls	6–9 years old	Case-control study	Higher urinary BPA concentrations linked to increased risk of ICPP
Jung et al., 2019 [105]	47 Korean girls with ICPP + 47 healthy controls	5–12 years old	Case-control study	No significant difference in urinary BPA levels between ICPP and controls
Bigambo et al., 2020 [106]	4737 girls	-	Meta-analysis	Significant link between 2,5-dichlorophenol exposure and earlier puberty; no significant association between earlier puberty and bisphenol A, triclosan, and benzophenone 3
Bigambo et al., 2023 [90]	21 Chinese girls with ICPP + 149 healthy girls	2–10 years old	Case-control study	BPA substitutes linked to higher risk of precocious puberty in girls
Huynh et al., 2024 [112]	124 Vietnamese children with ICPP and 126 healthy controls	-	Case-control study	Higher urinary BPA levels in children with precocious puberty
**Phthalates and Obesity**				
Buser et al., 2014 [125]	100	6–19 years	Cross-sectional study	Urinary concentrations of LMW phthalate metabolites are linked to higher obesity rates in male children and adolescents
Deierlein et al., 2016 [130]	1239	6–8 years	Longitudinal study	The results showed that LMW PAEs (MEP, MBP, and MiBP) were positively associated with increased BMI and waist circumference scores in these girls
Bulus et al., 2016 [101]	134	7–8 years	Case-control study	Higher phthalate levels in girls with CPP suggest that phthalates might impact the central nervous system and trigger puberty-related pathways
Srilanchakon et al., 2017 [131]	136	7–9 years	Cross-sectional study	Girls with precocious puberty had an association with increased MEP concentration. This is the first report of the association between urinary phthalate levels and precocious puberty in Thai girls
Hashemipour et al., 2018 [132]	150	7–9 years	Case-control study	Diethyl hexyl phthalate metabolites (MEHP, 5OH-MEHP, and 5oxo-MEHP) in girls with precocious puberty were significantly higher than those in the control group, indicating the possible role of these metabolites as endocrine-disrupting agents, in particular in the reproductive system
Amin et al., 2018 [133] 7/21/24 8:16:00 a.m.	242	6–18 years	Cross-sectional study	Urinary PAE levels of MBzP, MBP, MMP, MEHP, and MEHHP were significantly associated with childhood obesity. Additionally, MBzP and MEHP were related to triglyceride levels and obesity
Xia et al., 2018 [134]	149	10–15 years	Case-control study	Phthalate exposure might contribute to the development of high weight and obesity in school-age children
Wang et al., 2022 [128]	798	7–10 years	Cross-sectional study	The level of PAE metabolite exposure was linked to the risk of abdominal obesity in Chinese students aged 7–10 years
Dong et al., 2022 [126]	2298	7–13 years	Case-control study	The results suggested that children in Xiamen City, China, were widely exposed to environmental PAE pollutants. Furthermore, this high exposure could increase the risk of high weight and obesity, particularly in girls
Su et al., 2023 [135]	220	2–14 years	Cohort study	Phthalate exposure at certain times may affect children’s reproductive development during puberty
Li et al., 2023 [127]	480	6–8 years	Case-control study	Diet and physical activity, but not phthalate metabolites, were associated with childhood obesity
Huynh et al., 2024 [112]	250	6–8 years	Case-control study	This study found BPA-glucuronide in 11.3% of the PP group but not in the control group, suggesting a potential link. The PP group also had a higher prevalence of MBP (8.1%) compared to the control group (2.4%)

BPA = bisphenol A; BMI = body mass index; ICPP = idiopathic central precocious puberty; IPPP = idiopathic peripheral precocious puberty; LMW = low molecular weight; PP = precocious puberty.

## 4. Limits

We acknowledge some limitations of this review. Firstly, it is a narrative review, and the non-systematic nature of such reviews means there are no formally established guidelines for their conduct, potentially introducing biases in selection. For instance, we considered only articles published on PubMed, but there are also other databases and search engines; thus, it is possible that some studies were not included in this review.

Additionally, the literature shows inconsistent results regarding EDC levels and dietary patterns. Stronger, longitudinal studies are needed to establish clearer links. In addition, studies often focus on specific populations, making it difficult to generalize results globally. Therefore, comprehensive geographic and demographic studies are needed.

Another limitation is the difficulty in accurately measuring dietary exposure to EDCs due to variations in individual dietary habits, food preparation methods, and contamination levels in different food sources. Understanding the bioavailability and metabolism of EDCs in different individuals is also complex. Factors such as age, gender, and genetic variations may influence how these chemicals are metabolized in the body.

Finally, external contamination during sample collection, storage, or processing can alter results, making it difficult to obtain accurate exposure measurements.

Therefore, although evidence links phthalates to early puberty and obesity, further research is needed to clarify these relationships and better understand their correlation.

## 5. Conclusions

This review demonstrates that EDCs, such as phthalates and BPA, are linked to various health issues, including obesity and metabolic and endocrinological disorders like precocious puberty.

The diverse outcomes observed across different studies highlight the complexity of the effects of phthalates and BPA on the human body, both in terms of early puberty, particularly in girls, and obesity, with its metabolic disruptions. Moreover, obesity, which is independently associated with early puberty, might confound the relationship between EDC exposure and pubertal timing.

Dietary intake, particularly the consumption of processed and packaged foods, is a primary route of exposure to these chemicals. A useful starting point would be to minimize their consumption, as they often contain higher levels of phthalates and BPA. Instead, priority should be given to fresh and minimally processed foods, such as fresh fruit, vegetables (preferably not packaged), whole grains, and lean proteins. This diet promotes the Mediterranean Diet, which can help reduce exposure to EDCs and support overall health.

Additionally, the broader environmental context, including biodiversity and planetary health, plays a critical role in addressing the impacts of EDCs. Integrated approaches that emphasize the conservation of biodiversity and the sustainable management of natural resources are essential for mitigating these risks [136].

The existing body of evidence underscores the need for more rigorous and comprehensive research to better understand the role of phthalates and BPAs in pubertal development and obesity. Longitudinal studies with larger sample sizes, standardized exposure assessment protocols, and consideration of confounding factors such as BMI and metabolic health are essential to provide more definitive answers. Additionally, there is a pressing need to explore the mechanisms through which phthalates and BPAs exert their effects, including potential epigenetic changes and interactions with other environmental chemicals.

Given the significant public health implications, it is essential to adopt a precautionary approach by minimizing exposure to these EDCs, particularly in vulnerable populations like children and pregnant women. In light of these findings, public health strategies should focus on reducing exposure to EDCs through diet. One effective approach could involve increasing transparency in food labeling by not only indicating the presence of BPA but also providing quantifiable amounts. This would empower consumers, particularly those in vulnerable groups such as children and pregnant women, to make more informed decisions and consciously limit their exposure to BPA, thereby reducing potential health risks associated with these chemicals.

Collaboration among policymakers, healthcare providers, and researchers is essential to deepen our understanding of these chemicals and to develop effective strategies for reducing their presence in consumer products and the environment.

By tackling these challenges, we can safeguard future generations from the harmful effects of endocrine-disrupting chemicals.
